# A Genomotaxonomy View of the *Bradyrhizobium* Genus

**DOI:** 10.3389/fmicb.2019.01334

**Published:** 2019-06-13

**Authors:** Ernesto Ormeño-Orrillo, Esperanza Martínez-Romero

**Affiliations:** ^1^Laboratorio de Ecología Microbiana y Biotecnología, Departamento de Biología, Facultad de Ciencias, Universidad Nacional Agraria La Molina, Lima, Peru; ^2^Centro de Ciencias Genómicas, Universidad Nacional Autónoma de México, Cuernavaca, Mexico

**Keywords:** nodulation, nitrogen-fixation, phylogenomics, legume symbionts, *Rhodopseudomonas*

## Abstract

Whole genome analysis of the *Bradyrhizobium* genus using average nucleotide identity (ANI) and phylogenomics showed the genus to be essentially monophyletic with seven robust groups within this taxon that includes nitrogen-fixing nodule forming bacteria as well as free living strains. Despite the wide genetic diversity of these bacteria no indication was found to suggest that the *Bradyrhizobium* genus have to split in different taxa. Bradyrhizobia have larger genomes than other genera of the Bradyrhizobiaceae family, probably reflecting their metabolic diversity and different lifestyles. Few plasmids in the sequenced strains were revealed from *rep* gene analysis and a relatively low proportion of the genome is devoted to mobile genetic elements. Sequence diversity of *recA* and *glnII* gene metadata was used to theoretically estimate the number of existing species and to predict how many would exist. There may be many more species than those presently described with predictions of around 800 species in nature. Different arguments are presented suggesting that nodulation might have arose in the ancestral genus *Bradyrhizobium*.

## Introduction

*Bradyrhizobium* is one of the several genera of nitrogen fixing bacteria capable of forming symbiotic nodules in legumes. *Bradyrhizobium* strains were previously designated the slow growing *Rhizobium* and recognized as an independent genus in 1982 (Jordan, 1982). The number of species in *Bradyrhizobium* has increased largely in recent years^1^. Notably, there are photosynthetic bacteria among bradyrhizobia (Ladha and So, 1994) which need no Nod factors to induce nodules (Giraud et al., 2007) and this opened a new research area on plant nodulation. Non-symbiotic bradyrhizobia are dominant in forest soils (VanInsberghe et al., 2015).

Different *Bradyrhizobium* species are the main nodule symbionts of important crop legumes such as soybean (Xu et al., 1995; Delamuta et al., 2013), Lima bean (Durán et al., 2014) or peanuts (Steenkamp et al., 2008) and have been isolated from nodules of many tropical (Ramírez-Bahena et al., 2009; López-López et al., 2013; Delamuta et al., 2015) and temperate legumes (Vinuesa et al., 2005a; Stepkowski et al., 2007). The ancestral symbionts in *Phaseolus* could have been bradyrhizobia (Servín-Garcidueñas et al., 2014) with a later symbiont shift to *Rhizobium* in nodules of some temperate *Phaseolus* species.

A large diversity of bradyrhizobia has been revealed with the sequence of few genes used as molecular markers. Within bradyrhizobia, 16S rRNA genes are known not to provide adequate sequence diversity to clearly recognize distinct species (Willems et al., 2001). For this, markers such as *recA, glnII, atpD*, *dnaK, gyrB*, and *rpoB*, have been frequently used to characterize bradyrhizobial species and strains (Vinuesa et al., 2005b; Rivas et al., 2009; Delamuta et al., 2012). Novel markers such as *ftsA* gene provide congruent phylogenies to those derived from *recA* and *glnII* gene sequences (Kalita and Malek, 2019). *Bradyrhizobium* diversity knowledge has expanded recently with studies from native legumes in Africa (Gronemeyer et al., 2017; Jaiswal and Dakora, 2019; Puozaa et al., 2019), from Brazilian and Indian *Chamaecrista* (Santos et al., 2017; Rathi et al., 2018), from threatened native species in Brazil (Fonseca et al., 2012), from Genisteae plants in Poland (Kalita and Malek, 2017), indigenous trees in China (Yao et al., 2015) as examples. In addition, new bradyrhizobial species have been reported as well (Yao et al., 2015; Araujo et al., 2017; Gronemeyer et al., 2017; Helene et al., 2017; Ahnia et al., 2018; Bunger et al., 2018).

Additionally, some bradyrhizobial strains are capable of fixing nitrogen as endophytes of some plants (Piromyou et al., 2015). Growth-independent approaches have shown a broad distribution of bradyrhizobia associated with roots of many non-legume plants such as rice, maize and pines (Chaintreuil et al., 2000; Tan et al., 2001) and bradyrhizobia have been found in plant tumors (Rivas et al., 2004; Islam et al., 2008) and also in earthworm (Thakuria et al., 2010) and insect guts (Degli Esposti and Martinez Romero, 2017).

Nowadays there are publicly available genomes of 187 bradyrhizobial strains. Bradyrhizobia have characteristic large genomes with few plasmids and no symbiosis plasmids with one exception (Okazaki et al., 2015). In *Bradyrhizobium* chromosomes there are symbiosis islands which carry *nod* and *nif* genes that are responsible for nodulation and nitrogen fixation, respectively. Symbiosis markers that are commonly used toward symbiosis phylogenetic reconstructions are *nifH* and *nod* genes. In regard to nodulation genes, there is a large diversity of *nodA* or *nodC* genes (Stepkowski et al., 2007; Martínez-Romero et al., 2010) and the phylogenies from these genes support a vertical and also a horizontal transfer of these genes among bradyrhizobia (Moulin et al., 2004; Menna and Hungria, 2011). Symbiovars in relation to host specificity have been identified for some bradyrhizobial species (Rogel et al., 2011) and novel symbiovars have been described (Bejarano et al., 2014; Cobo-Diaz et al., 2014; Ramirez-Bahena et al., 2016; Delamuta et al., 2017; Salmi et al., 2018; Martins da Costa et al., 2019).

Novel metrics to recognize species are based on genome analysis and average nucleotide identity, ANI (Richter and Rossello-Mora, 2009) and phylogenomics (Wu and Scott, 2012) are proving to be very useful toward this goal. A phylogenomic study of *Bradyrhizobium* strains showed that the presence and type of flagellum are phylogenetically determined (Garrido-Sanz et al., 2019). Other phylogenomic-based studies have been reported for the beta-rhizobia (Beukes et al., 2017; Estrada-de Los Santos et al., 2018) highlighting the existence of novel genera. It is the aim of this work to use a similar genomic-based approach and present an up-dated global genomic-based analysis of the *Bradyrhizobium* genus to further support its taxonomic status. Previously, such a study was performed for *Rhizobium, Sinorhizobium* and *Agrobacterium*, shedding light on these genera taxonomical designations (Ormeño-Orrillo et al., 2015).

## Materials and Methods

### Phylogenomic Analysis

All *Bradyrhizobium* genome sequences available in GenBank as of December 2018 were retrieved. An all-versus-all average nucleotide identity (ANI) matrix was constructed using OrthoANI (Lee et al., 2016). Non-metric multidimensional analysis on the ANI matrix was performed with PAST to identify outlier genome sequences. Species-level clusters were defined at a 95% ANI cutoff value and representative genomes from each cluster were selected for further analysis. For the phylogenomic reconstruction, Prodigal was used for *de novo* prediction of protein-coding genes for each selected genome. Amino acid sequences of 31 conserved phylogenetic markers were retrieved from each genome using AMPHORA2 (Wu and Scott, 2012) and aligned with muscle (Edgar, 2004). Each alignment was processed with trimAl to identify and remove poorly aligned regions (Capella-Gutierrez et al., 2009). All alignments were concatenated and a maximum likelihood phylogeny was constructed with PhyML (Guindon et al., 2010). Tree node support was evaluated with bootstrap analysis of 1000 replicates.

### Mobilome Analysis

The presence of plasmid replication systems in the sequenced genomes was evaluated by looking for homologs to the replication protein B (RepB) using BLASTP searches against a database of all the *Bradyrhizobium* proteins obtained in the previous section. The number and size of genomic islands in complete and almost complete genomes were determined with the IslandViewer 4 server (Bertelli et al., 2017).

### Species Richness Analysis

*Bradyrhizobium* nucleotide sequences from the recombinase A protein (*recA*) and glutamine synthetase type II (*glnII*) genes were retrieved from the GenBank database and aligned using MAFFT (Katoh and Standley, 2013). The multiple sequence alignments were trimmed with Bioedit (Hall, 1999) recovering 336 or 454 bp fragments common to most *recA* or *glnII* sequences, respectively, shorter sequences were discarded. An all-versus-all identity distance matrix was constructed from the alignments with the dist.seqs command of mothur (Schloss et al., 2009). Sequences were classified into species-level OTUs with the cluster command of mothur. OTU abundances were used to calculate species richness estimators with SpadesR^2^ and EstimateS^3^, and to construct rarefaction curves with PAST (Hammer et al., 2001).

## Results and Discussion

### *Bradyrhizobium* Genomes in GenBank

**FIGURE 1:**
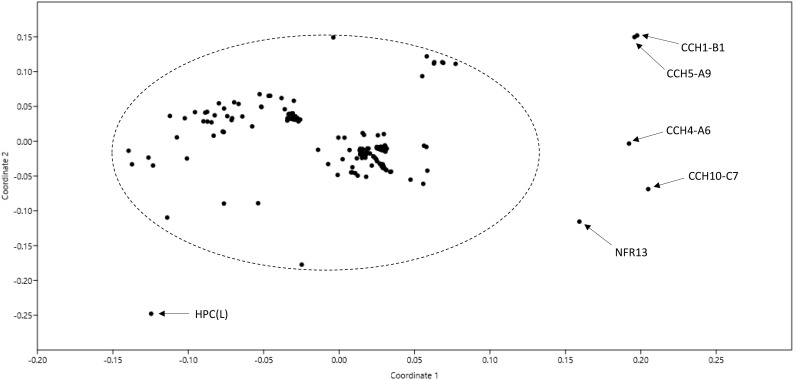
Non-metric multidimensional scaling (nMDS) of pair-wise ANI values of 187 genomes named as *Bradyrhizobium* in GenBank as of December 2018. The ellipse includes genomes of *bona fide* bradyrhizobia. Names of non-bradyrhizobial outlier strains are indicated.

One hundred and eighty-seven genomes of strains named as *Bradyrhizobium* were available in GenBank at the time of writing this manuscript in December 2018 (Supplementary Table 1). A non-metric multidimensional scaling graph of ANI values shared by these genomes allowed the identification of a core set of 180 related genomes plus 7 outliers (Figure 1). Upon examination of selected phylogenetic markers (*rrs*, *recA*, and/or *glnII*), only the 180 related genomes corresponded to *bona fide* bradyrhizobia. The outliers were misnamed strains belonging to other genera (see Supplementary Table 1 for details).

Based on a 95% cutoff ANI value, the 180 *bona fide* bradyrhizobial genomes in GenBank were grouped into 93 species-level clusters (referred here as OTUs), the majority of which (72%) were represented by only one sequenced strain (Table 1). Twenty nine type strains were present among the 180 sequenced bradyrhizobia. The most represented species were *Bradyrhizobium japonicum* and *Bradyrhizobium canariense*, each with 15 strains; followed by *B. diazoefficiens* and *B elkanii* with 9 and 8 strains, respectively. The abundance of sequences from species able to nodulate soybean indicates the bias toward genomic studies directed at symbionts from this agronomical important legume.

**Table 1:** Classification of 180 *Bradyrhizobium* genome-sequenced strains into superclades, OTUs and species.

Strains	Superclade^a^	OTU^b^	Species^c^
USDA 6^T∗^, CCBAU 15354, CCBAU 15517, CCBAU 15618, CCBAU 25435, CCBAU 83623, USDA 38, USDA 123, SEMIA 5079, Is-34, E109, FN1, J5, G22	I	1	*B. japonicum*
WSM4349, UBMA050, UBMA051, UBMA060, UBMA052, UBMA061, UBMA195, UBMAN05, UBMA122, UBMA182, UBMA192, UBMA183, UBMA510, UBMA181, UBMA171	I	2	*B. canariense*
USDA 110^T∗^, CCBAU 41267, USDA 122^∗^, SEMIA 5080, Is-1, NK6, Y21	I	3	*B. diazoefficiens*
OO99^T^, CCBAU 15544, CCBAU 15615, CCBAU 15635, USDA 4, L2	I	4	*B. ottawaense*
CCBAU 10071^T^, CCBAU 05623, CCBAU 25021, CCBAU 35157, BR3267, SUTN9-2	I	5	*B. yuanmingense*
WSM2254, JGI 0001019-J21, cf659	I	6	
CCBAU 05525, CCBAU 83689, USDA 135	I	7	
LMG 26795^T^, USDA 3384, CB756	I	8	*B. arachidis*
is5, in8p8, Leaf396	I	9	
WSM1417, URHA0013	I	10	
WSM2793, Rc3b	I	11	
CCGE-LA001, DOA1	I	12	
ERR11^T^, AC87j1	I	13	*B. shewense*
WSM471, BF49_genome1	I	14	
BR 10247^T^, Cp5.3	I	15	*B. neotropicale*
LTSP849, LTSP857	I	16	
OK095	I	17	
Y36	I	18	
S23321	I	19	
TSA1^T^	I	20	*B. nitroreducens*
39S1MB	I	21	
UBMA197	I	22	
Ec3.3	I	23	
JGI 0001019-M21	I	24	
Ghvi	I	25	
INPA54B^T^	I	26	*B. forestalis*
85S1MB	I	27	
DOA9	I	28	
CCH5-F6	I	29	
BR 10245^T^	I	30	*B. centrolobii*
22	I	31	
CCNWSX0360	I	32	
WSM3983	I	33	
CCBAU 43298	I	34	
NAS80.1	I	35	
Rc2d	I	36	
YR681	I	37	
WSM1253	I	38	
BR10280^T^	I	39	*B. sacchari*
JGI 0001002-A22	I	40	
BR 446^T^	I	41	*B. stylosanthis*
Gha	I	42	
USDA 124	I	43	
MOS002	I	44	
AS23.2	I	45	
BR3351^T^	I	46	*B. manausense*
WSM1743	I	47	
AT1	I	48	
MOS003	I	49	
USDA 76^T^, 587, CCBAU 05737, CCBAU 43297, USDA 94, BLY6-1, BLY3-8, TnphoA 33	II	50	*B. elkanii*
PAC 48^T^, USDA 3254, USDA 3259, BR3262, UFLA 03-321, R5	II	51	*B. pachyrhizi*
OHSU_III, UASWS1015, UASWS1016, UBA2491, SK17	II	52	
DFCI-1, 17-4 str. JCM 18382, PARBB1, MOS004	II	53	
SEMIA 690^T^, UFLA03-84	II	54	*B. viridifuturi*
LTSPM299, LTSP885	II	55	
MT12	II	56	
SEMIA 6208^T^	II	57	*B. embrapense*
SEMIA 6148^T^	II	58	*B. tropiciagri*
BR 10303^T^	II	59	*B. macuxiense*
C9	II	60	
NAS96.2	II	61	
SEMIA 6399^T^	II	62	*B. mercantei*
th.b2	II	63	
ORS 285^∗^	III	64	
ORS 375	III	65	
STM 3809	III	66	
BTAi1	III	67	
STM 3843	III	68	
S58^T^	III	69	*B. oligotrophicum*
ORS 278	III	70	
GAS524, GAS522, MT34	IV	71	
RST89^T^, RST91	IV	72	*B. algeriense*
LmjM3^T^, LmjM6	IV	73	*B. valentinum*
URHA0002	IV	74	
LMTR 21^T^	IV	75	*B. paxllaeri*
PAC68^T^	IV	76	*B. jicamae*
URHD0069	IV	77	
CCBAU 23086^T^	IV	78	*B. lablabi*
GAS138	IV	79	
GAS165	IV	80	
GAS478	IV	81	
GAS242	IV	82	
GAS499	IV	83	
LMTR 3	IV	84	
WSM1741	IV	85	
GAS369	IV	86	
LMTR 13^T^	IV	87	*B. icense*
Ro19^T^	IV	88	*B. retamae*
ARR65	V	89	
Tv2a-2	V	90	
Ai1a-2	VI	91	
WSM2783	VI	92	
GAS401	VII	93	

*^a^Based on a phylogenic analysis performed with AMPHORA2. ^b^Based on a 95% cutoff ANI value. ^c^Based on a genome-sequenced type or reference strain. ^∗^Two genomes are available for this strain.*

### Phylogenomic Relationships in the Bradyrhizobiaceae

**FIGURE 2:**
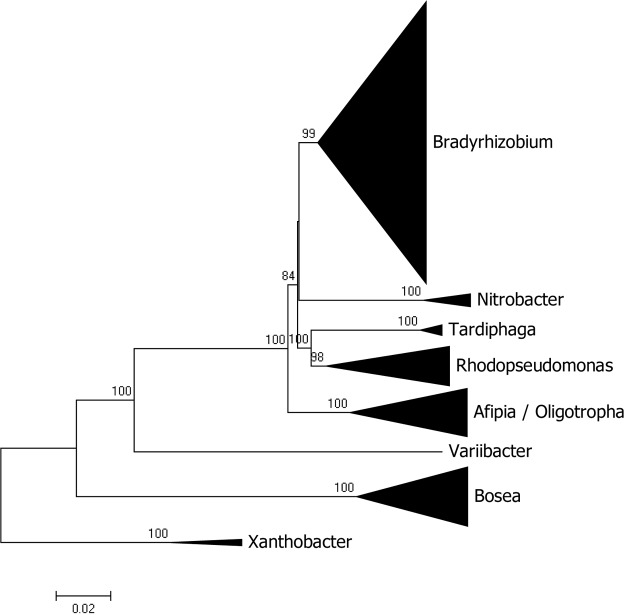
Phylogenomic tree of Bradyrhizobiaceae rooted with genomes of *Xanthobacter*. The tree was constructed with maximum likelihood using a concatenated alignment of 31 conserved proteins identified with AMPHORA2. Bootstrap node support values lower than 70% are not shown.

Besides *Bradyrhizobium*, other genera of the Bradyrhizobiaceae family with sequenced genomes are *Bosea*, *Afipia*, *Rhodopseudomonas*, *Nitrobacter*, *Tardiphaga*, *Oligotropha*, and *Variibacter* with 30, 24, 18, 6, 4, 3, and 1 sequences, respectively. A phylogenomic analysis of the family showed that most genera segregated as expected with the sole exception of *Oligotropha* and *Afipia* which intermingle (see Figure 2 for a condensed tree and Supplementary Figure 1 for a full tree). *Bosea* was the most distantly related genus in the family and its clustering with the other genera was not significantly supported by bootstrap analysis.

### Intrageneric Structure of the Genus *Bradyrhizobium*

**FIGURE 3:**
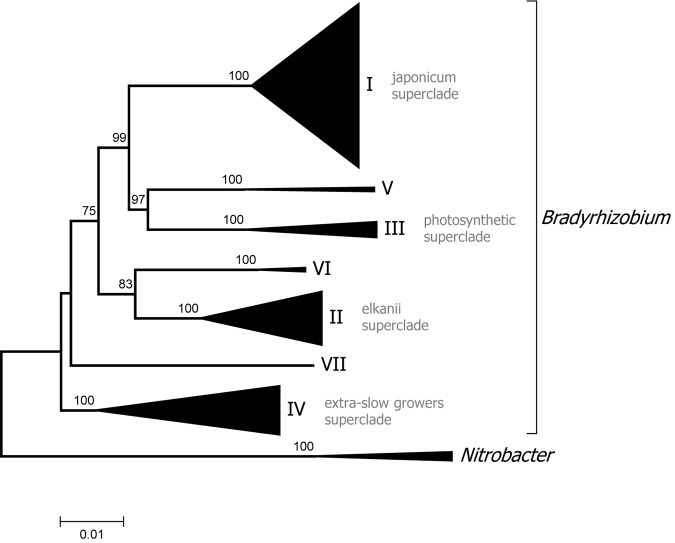
Phylogenomic tree of *Bradyrhizobium*. Superclades are indicated with Roman numerals. The tree was constructed with maximum likelihood using a concatenated alignment of 31 conserved proteins identified with AMPHORA2 (7672 aa alignment length). Bootstrap node support values lower than 70% are not shown.

Two *Bradyrhizobium* superclades are recognized based on analysis of the 16S rDNA gene (Willems et al., 2001; Ormeño-Orrillo et al., 2006). Superclade I includes *B. japonicum* and related species while superclade II contains *B. elkanii* and allied species. The new phylogenomic analysis supports the existence of those clades but revealed a more complex structure within *Bradyrhizobium* with additional groups (see Figure 3 for a condensed tree and Supplementary Figure 2 for a full tree).

Superclade III encompassed several photosynthetic strains isolated from *Aeschynomene indica*, as well as *Bradyrhizobium oligotrophicum* which also forms nodules in that legume (Okubo et al., 2013). *Bradyrhizobium denitrificans*, which comprise photosynthetic bacteria able to nodulate *A. indica* (Van Berkum et al., 2006), also belong to superclade III as shown by MLSA analysis (Ramírez-Bahena et al., 2013). Members of superclade III were the first to be recognized as having the capacity to induce nodulation in the absence of the canonical *nod* genes (Giraud et al., 2007).

Superclade IV includes strains isolated from several cultivated and wild legumes such as *Phaseolus lunatus* and *Lupinus maria-josephi*. Bacteria from this superclade closely group with *B. elkanii* in 16S rRNA phylogenies but its distinctiveness was first recognized by *dnaK* sequence analysis (Ormeño-Orrillo et al., 2006) and MLSA (Sanchez-Cañizares et al., 2011) and is now confirmed by phylogenomics. These bradyrhizobia have the characteristics of extra slow growth forming punctate colonies on YEM medium and usually strong alkali production.

Superclade V was formed by only two genomes one of them from strain Tv2a.2 from *Tachigali versicolor*, that was previously shown to occupy instable positions in the *Bradyrhizobium* phylogeny depending on the marker used (Parker, 2000). Superclade VI also grouped only two genomes including that of strain Ai1a.2 a representative of neotropical bradyrhizobia which possess a characteristic insertion in their 23S ribosomal gene sequence (Qian et al., 2003). Finally, superclade VII included a single strain, GAS401, which was isolated from a forest soil in the United States.

### Several Genera Inside *Bradyrhizobium*?

The wide genotypic and phenotypic diversity within *Bradyrhizobium* may suggest that it includes several genera. As early as 1990, strains now classified in superclade IV, were proposed to constitute the separate genus *Photorhizobium* because of their photosynthetic abilities and induction of stem nodulation (Eaglesham et al., 1990; Ladha and So, 1994). The most up to date, although not officially recognized, identity threshold for genus circumscription based on the 16S rRNA gene is 96.4% (Yarza et al., 2014). When applied to bradyrhizobia, superclade II strains are different enough to be considered a separate genus, however, the same threshold indicates that *Bradyrhizobium*, *Rhodopseudomonas*, *Nitrobacter*, and *Afipia* may constitute a single genus (Willems et al., 2001). Superclade II strains possess recombinant segments in their 16S ribosomal gene that explains their sequence divergence from other bradyrhizobia (Van Berkum et al., 2003) but this case of localized recombination with other bacteria do not justify their separation from the genus.

Recently, Qin et al. (2014) used the percentage of conserved proteins (POCP) as a genome metric for genus circumscription. According to their proposal, two bacteria may belong to the same genus if they share 50% or more of their proteins. In the case of *Bradyrhizobium*, all compared strains share >50% of their protein complements supporting a single genus. Different genera of the Bradyrhizobiaceae family had POCPs values between 40 and 50% except for most comparisons between bradyrhizobia and *Rhodopseudomonas* that can share more than half of their proteins. Thus, in general the POCP metric supports *Bradyrhizobium* as a single genus but indicate a close relationship with *Rhodopseudomonas.* Conservation of gene content may indicate not only phylogenetic relationship but also phenotypic similarity (Martín et al., 2003). Both *Bradyrhizobium* and *Rhodopseudomonas* include nitrogen fixing and photosynthetic strains, and, recently, a study suggest that *Rhodopseudomonas* can be mutualistic symbionts of some fungi (Le Roux et al., 2016). It will be worth to further explore the common features of both genera based on their genomic sequences.

### Genome Size in *Bradyrhizobium*

**FIGURE 4:**
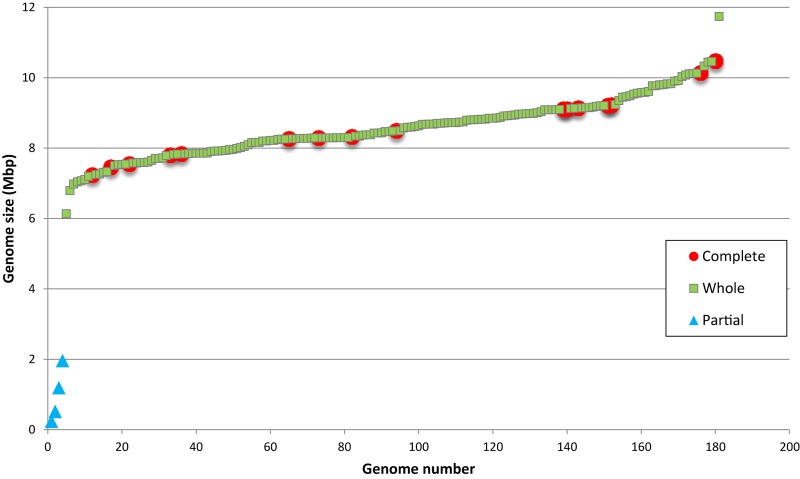
Size distribution of 180 genome assemblies of *Bradyrhizobium* strains.

Bradyrhizobia are considered bacteria with large genomes (Kundig et al., 1993), however the range of genome sizes in the genus is still unknown. A graphic showing the size distribution of the 180 genome assemblies of *Bradyrhizobium* strains is shown in Figure 4. Since only 15 of the genome sequences correspond to strains with closed replicon(s), we determined if the remaining sequences represent partial or whole genomes. A completeness analysis with BUSCO revealed that the four smaller assemblies, all <2 Mbp, represented partially sequenced genomes. Information available from those genomes revealed that they are derived from single cells or metagenomes likely explaining their incompleteness.

The smallest sequence representing a complete genome, was 6.1 Mbp in size and corresponded to strain GAS165 isolated from a forest soil. This size is similar to that found in *Rhizobium* and *Sinorhizobium* genomes. The largest complete assembly (11.7 Mbp) was also from an isolate obtained from soil (strain GAS478). Interestingly, both GAS 165 and GAS478 strains which belong to superclade IV, lacked symbiosis genes which may indicate that genome size in *Bradyrhizobium* is not related to its ability to engage in symbiosis with legumes. It is worth noting that the largest bradyrhizobial genome is close in size to that of *Sorangium cellulosum* (13 Mbp) the bacteria with the largest known genome (Schneiker et al., 2007).

**FIGURE 5:**
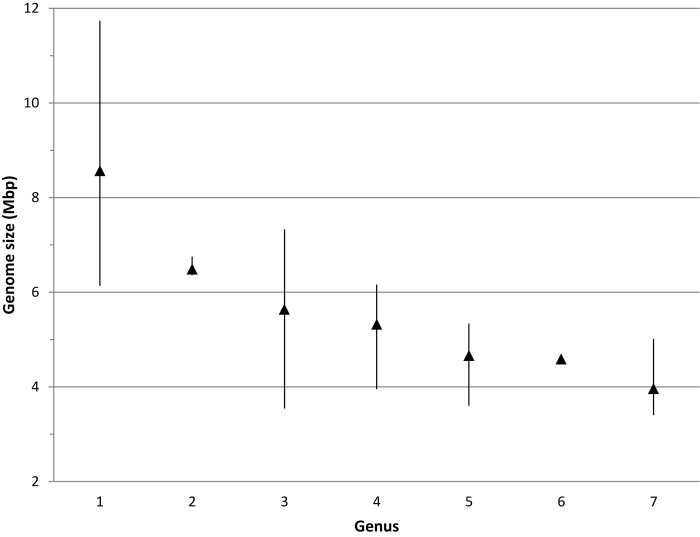
Genome size range in the different genera of the Bradyrhizobiaceae family. 1, *Bradyrhizobium*; 2, *Tardiphaga*; 3, *Bosea*; 4, *Rhodopseudomonas*; 5, *Afipia*/*Oligotropha*; 6, *Variibacter*; 7, *Nitrobacter*. Triangles indicate the mean genome size.

Most *Bradyrhizobium* strains had genome sizes between 7 and 10 Mbp with a mean size of 8.6 Mbp (Figure 4). When compared to other genera in its family, bradyrhizobia have the largest genomes (Figure 5) followed by *Tardiphaga* and *Bosea*, while the smallest genomes were those from *Nitrobacter*. The three genera with the biggest genomes can interact with plants (De Meyer et al., 2012; De Meyer and Willems, 2012). On the other hand the smallest genomes are found in metabolically limited bacteria like *Nitrobacter*, *Oligotropha* or *Variibacter* or in intracellular pathogens like *Afipia*. Thus, genome size in the Bradyrhizobiaceae seems to be related to lifestyle.

### The *Bradyrhizobium* Mobilome

Given the large genome size in bradyrhizobia we wondered which the proportion of plasmids and genomic islands (GIs) was. Bradyrhizobia are typically regarded as unireplicon bacteria, however, strains with plasmids have been reported (Cytryn et al., 2008) including a single case of a symbiosis plasmid (Okazaki et al., 2015). The presence of extra-chromosomal replicons was evaluated in the genome-sequenced strains by searching for genes coding for homologs of the plasmid partitioning protein RepB. We found *repB* genes in 35 genomes (Supplementary Table 2) with almost half of the strains (*n* = 17) possessing a single homolog which suggested the presence of a single plasmid. The remaining strains may harbor up to 6 plasmids, although it should be noted that a single plasmid could harbor two *repABC* operons. The few completely-sequenced plasmids ranged in size from 136 to 285 kbp which is a size range similar to that found by pulse-field gel electrophoresis analysis of plasmids in a diverse population of *Bradyrhizobium* (Cytryn et al., 2008). Plasmids can represent from 2.7 to 6.6% of the genome in a single strain. The sequence of the single *Bradyrhizobium* symbiosis plasmid known to date was reported as a scaffold that is larger than other plasmids in the genus (736 kbp) and which represents 9.4% of the corresponding genome (Okazaki et al., 2015).

Mobile elements in unireplicon bacteria are typically present as clusters of genes known as genomic islands (GIs) (Juhas et al., 2009). As mutualistic symbionts of legumes, bradyrhizobia typically possess nodulation and nitrogen fixation genes grouped in a symbiosis island (SI) (Kaneko et al., 2002). The percentage of a *Bradyrhizobium* chromosome devoted to GIs was calculated using the Island Viewer 4 server applied on completely sequenced genomes (Table 2). It was found that from 5.2% and up to 17.8% of the chromosome can be regarded as the GI mobilome of individual strains.

**Table 2:** Percentage of genomic islands (GI) in the chromosomes and genomes of *Bradyrhizobium.*

Organism	Genome size (bp)	Chromosome (bp)	GI (bp)	GI/genome (%)	GI/chromosome (%)
*Bradyrhizobium diazoefficiens* USDA110^T^	9,105,828	9,105,828	1,616,869	17.8	17.8
*Bradyrhizobium* sp. BTAi1	8,493,513	8,264,687	946,881	11.1	11.5
*Bradyrhizobium* sp. ORS 278	7,456,587	7,456,587	736,932	9.9	9.9
*Bradyrhizobium* sp. S23321	7,231,841	7,231,841	377,879	5.2	5.2
*Bradyrhizobium japonicum* USDA 6^T^	9,207,384	9,207,384	1,618,905	17.6	17.6
*Bradyrhizobium* sp. CCGE-LA001	7,833,499	7,833,499	992,465	12.7	12.7
*Bradyrhizobium oligotrophicum* S58	8,264,165	8,264,165	930,097	11.3	11.3
*Bradyrhizobium japonicum* E109	9,224,208	9,224,208	1,573,528	17.1	17.1
*Bradyrhizobium diazoefficiens* NK6	10,475,157	9,780,023	1,491,477	14.2	15.3
*Bradyrhizobium icense* LMTR 13	8,322,773	8,322,773	705,512	8.5	8.5
*Bradyrhizobium japonicum* J5	10,138,651	10,138,651	1,819,761	17.9	17.9
*Bradyrhizobium diazoefficiens* USDA 122	9,136,536	9,136,536	1,111,353	12.2	12.2
*Bradyrhizobium* sp. BF49_genome1	7,547,693	7,547,693	960,583	12.7	12.7
*Bradyrhizobium* sp. SK17	8,288,568	8003090	557625	6.7	7.0
*Bradyrhizobium* sp. ORS 285	7,797,098	7,797,098	796588	10.2	10.2

### How Many Species of *Bradyrhizobium* Would Be?

Another aspect related to the wide diversity found among bradyrhizobia is related to their species richness. Up to December 2018, forty one *Bradyrhizobium* species have been described. As previously mentioned, there would be 93 species among the genome-sequenced *Bradyrhizobium* strains, i.e., more than twice the number of presently described species. A quick survey of some of the studies published on *Bradyrhizobium* diversity in different regions like North America (Ormeño-Orrillo et al., 2012), South America (Delamuta et al., 2012), Africa (Aserse et al., 2012), Asia (Vinuesa et al., 2008), and Australia (Stepkowski et al., 2012) suggest that the number of bradyrhizobial species must be larger than a hundred but the total number is yet unknown.

To estimate the number of potential *Bradyrhizobium* species we decided to treat the 5678 *recA* and 3575 *glnII* sequences available in the GenBank database as meta-samples of the worldwide population of bradyrhizobia. Although other phylogenetic markers have also been used to characterize bradyrhizobia, numbers of their available sequences were much lower in comparison to *recA* and *glnII* (2411, 1771, 1758, 1465 for *dnaK*, *rpoB*, *atpD*, *gyrB*, respectively). We refrain to use the 16S rDNA gene despite having a large number of sequences in the databases because it is already known that sequences of this gene are too conserved in bradyrhizobia to discriminate between species and in some cases are even unable to discriminate between *Bradyrhizobium* and closely related genera (Willems et al., 2001).

**FIGURE 6:**
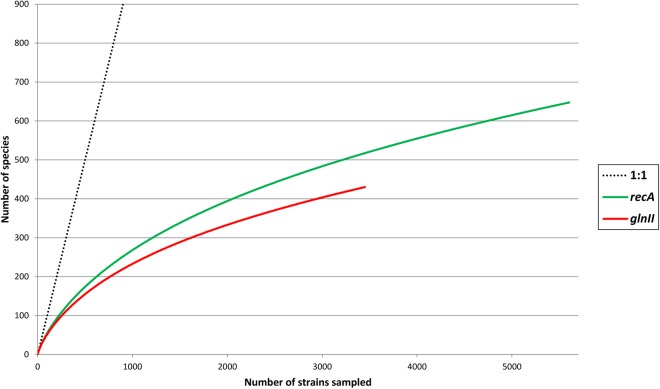
Rarefaction curves of species found in meta-samples of bradyrhizobia with sequenced *recA* or *glnII* genes. A hypothetical 1:1 line (each strain equal a new species) was drawn for reference to better represent the leveling of each meta-sample.

**FIGURE 7:**
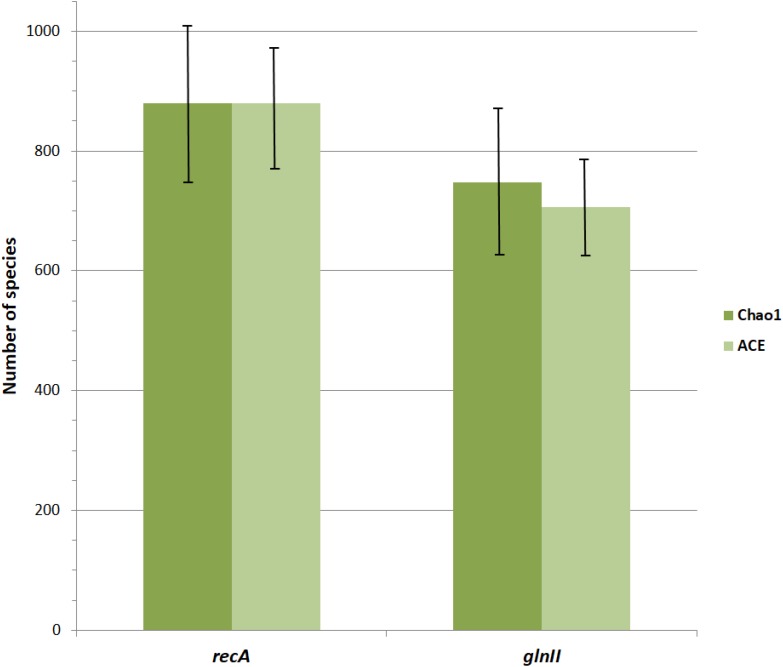
Chao1 and ACE non-parametric estimates of *Bradyrhizobium* species richness based on meta-samples of strains with sequenced *recA* or *glnII* genes. The *recA* metasample was rarefied to the number of *glnII* sequences (*n* = 3464). Vertical lines indicate standard errors.

Pair wise comparisons between sequences from *Bradyrhizobium* type strains revealed that nucleotide identities of 98.2% for *recA* and 98.8% for *glnII* can be used as cutoff values to discriminate between currently described bradyrhizobial species. Using those cutoff levels, the worldwide sampled bradyrhizobia with sequenced *recA* genes can be clustered into 648 species-level OTUs while *glnII* data revealed 431 potential species. Rarefaction curves of both meta-samples did not leveled off (Figure 6) suggesting that there are still species to be discovered. Non-parametric richness estimation (Chao, 2005) applied to both meta-samples indicated that there could be from 750 to 880 species of *Bradyrhizobium* in nature (Figure 7). It is worth noting that both meta-samples are composed primarily of root nodule isolates. Non-symbiotic bradyrhizobia inhabiting niches such as soil or rhizosphere, or as endophytes of non-legume plants may encompass additional species.

### *Bradyrhizobium*, the Mother of Nodule Symbiosis in Legumes?

The large genomic and phenomic diversity, as well as the high number of predicted species may indicate that the *Bradyrhizobium* genus is old. Interestingly, up till now this is the only nodule bacteria genus that contains photosynthetic bacteria and rhizobia that do not need Nod factors for nodulation. It is notable that it contains some very efficient strains for nitrogen fixation but also many generalists that form nodules in tropical legumes which in general are considered to precede temperate legumes. Several authors have noted that more “primitive” legume plants form nodules predominantly with *Bradyrhizobium*, thus lending support to the hypothesis that these rhizobia are their ancestral symbionts (Fonseca et al., 2012; Yao et al., 2014, 2015; Santos et al., 2017; Rathi et al., 2018). Similarly, in *Phaseolus* the ancestral symbionts were bradyrhizobia with a later symbiont shift to *Rhizobium* in nodules of some temperate species (Servín-Garcidueñas et al., 2014). The large diversity and number of different *nod* genes in bradyrhizobia (Martínez-Romero et al., 2010) may be suggestive that nodulation arose in bradyrhizobia. This has been a subject of discussion (Martínez-Romero, 1994; Parker, 2015; Sprent et al., 2017) with alternative views placing the origin of *nod* genes in beta-proteobacteria (Aoki et al., 2013) based on Nod factor exporter gene phylogenies. Since these exporters are not strictly required for nodulation (Cárdenas et al., 1996), we can suppose that accessory *nod* genes such as those for transporting Nod factors may be later additions after the emergence of common nodulation genes in *Bradyrhizobium*, then we may conciliate other possible origins for accessory *nod* genes.

During this article reviewing process, two papers that we must mention were published. The first one by Tindall (2019) stated that Bradyrhizobiaceae is an illegitimate name that needs to be replaced by Nitrobacteraceae because the latter contains *Nitrobacter* and was proposed earlier than Bradyrhizobiaceae and therefore takes precedence. Since this nomenclatural change did not affect our conclusions and because Bradyrhizobiaceae is how the family is still known, we choose to retain the name throughout this paper. However, we recognize that future studies will probably have to use Nitrobacteraceae as the proper family name. The second paper reported a phylogenomic analysis of bradyrhizobia and related taxa (Avontuur et al., 2019). Similar to our findings, Avontuur et al. (2019) confirmed the distinctiveness of the japonicum, elkanii and photosynthetic superclades and described additional infrageneric groups, albeit with some differences probably due to the use of different sets of genes for the phylogenomic reconstructions. In their analysis, our superclade IV was scattered in three groups named jicamae, soil 1 and soil 2; and our superclade VI was included in the elkanii group. In the first case we recognized that the larger number of genes used by Avontuur et al. (2019) in comparison to us, may have better resolved strains from our superclade IV, however, in the latter case we consider that superclade VI can be rightly segregated from the elkanii group by the presence of an insertion in their 23S ribosomal genes. Unlike us, Avontuur et al. (2019) tried to relate symbiotic and photosynthetic lifestyles with the infrageneric structure found within *Bradyrhizobium* but no clear relationship was found probably because those lifestyles are coded by accessory genes that can be gained and loss. On the other hand, our study includes aspects that were not covered by Avontuur et al. (2019) like species richness in *Bradyrhizobium* and the mobilome and putative plasmid content. We also provide a more thoroughly consideration of genome size in the Bradyrhizobiaceae and whether or not bradyrhizobia may be split into different genera. Thus, our analysis and those of Avontuur et al. (2019) provide complementary views on the highly diverse *Bradyrhizobium* genus.

## Author Contributions

EO-O and EM-R contributed conception and design of the study. EO-O performed the bioinformatics analysis. Both authors analyzed the results and wrote manuscript, and contributed to manuscript revision, read and approved the submitted version.

## Conflict of Interest Statement

The authors declare that the research was conducted in the absence of any commercial or financial relationships that could be construed as a potential conflict of interest.

## Footnotes

1 http://edzna.ccg.unam.mx/rhizobial-taxonomy/

2 https://chao.shinyapps.io/SpadeR/

3 http://viceroy.eeb.uconn.edu/estimates/
