# Supplementary material for: A Genomotaxonomy View of the Bradyrhizobium Genus
Source: Front Microbiol. 2019 Jun 13;10:1334. doi: 10.3389/fmicb.2019.01334 (PMC6585233; doi:10.3389/fmicb.2019.01334)

Supplementary Fig. 1

Phylogenomic tree of Bradyrhizobiaceae rooted with genomes of Xanthobacter. The tree was constructed with maximum likelihood using a concatenated alignment of 31 conserved proteins identified with AMPHORA2. Bootstrap node support values lower than 70% are not shown.

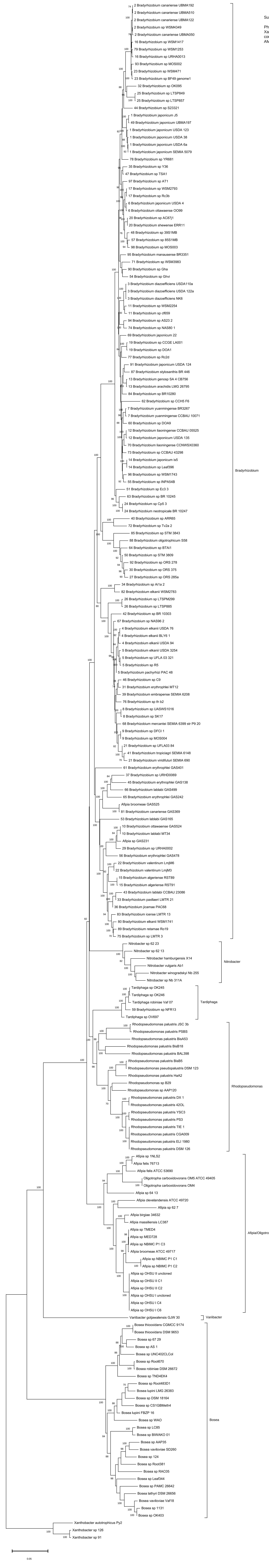

Supplement: Supplementary file 1 [file Image_1.pdf]
