# Supplementary material for: A Genomotaxonomy View of the Bradyrhizobium Genus
Source: Front Microbiol. 2019 Jun 13;10:1334. doi: 10.3389/fmicb.2019.01334 (PMC6585233; doi:10.3389/fmicb.2019.01334)
Supplement: Supplementary file 3 [file Table_1.pdf]

Supplementary Table 1. Genomes of strains labelled as *Bradyrhizobium* in GenBank

Strain	Accession	Superclade[a]	OTU[b]	Length (bp)
CCBAU 15354	GCA_000261545.1	I	1	10,126,594
CCBAU 15517	GCA_000261565.1	I	1	9,917,031
CCBAU 15618	GCA_000261585.1	I	1	9,824,012
CCBAU 25435	GCA_000261605.1	I	1	9,460,795
CCBAU 83623	GCA_000261625.1	I	1	10,074,298
USDA 6[T]	GCA_000284375.1	I	1	9,207,384
USDA 6[T]	GCA_000472985.1	I	1	9,131,141
USDA 38	GCA_000472745.1	I	1	9,608,975
USDA 123	GCA_000482525.1	I	1	10,457,665
SEMIA 5079	GCA_000661935.1	I	1	9,583,027
Is-34	GCA_000773865.1	I	1	10,326,597
E109	GCA_000807315.1	I	1	9,224,208
FN1	GCA_001038185.1	I	1	9,138,496
J5	GCA_001887695.1	I	1	10,138,651
G22	GCA_900011265.1	I	1	9,091,355
WSM4349	GCA_000373125.1	I	2	8,294,334
UBMA050	GCA_002108675.1	I	2	8,299,015
UBMA051	GCA_002108695.1	I	2	8,273,379
UBMA060	GCA_002108725.1	I	2	8,291,234
UBMA052	GCA_002108745.1	I	2	8,294,491
UBMA061	GCA_002108755.1	I	2	8,267,021
UBMA195	GCA_002108775.1	I	2	8,274,608
UBMAN05	GCA_002108805.1	I	2	8,376,411
UBMA122	GCA_002108825.1	I	2	8,214,782
UBMA182	GCA_002108845.1	I	2	8,216,640
UBMA192	GCA_002108865.1	I	2	8,287,509
UBMA183	GCA_002108875.1	I	2	8,288,770
UBMA510	GCA_002108905.1	I	2	8,367,860
UBMA181	GCA_002108965.1	I	2	8,299,676
UBMA171	GCA_002108995.1	I	2	8,279,565
USDA110[T]	GCA_000011365.1	I	3	9,105,828
USDA110[T]	GCA_001642675.1	I	3	9,106,064
CCBAU 41267	GCA_000261765.1	I	3	9,121,826
USDA 122	GCA_000473025.1	I	3	8,976,980
USDA 122	GCA_001908315.1	I	3	9,136,536
SEMIA 5080	GCA_000648595.2	I	3	9,085,533
Is-1	GCA_001280585.1	I	3	8,983,878
NK6	GCA_001549695.1	I	3	10,475,157
Y21	GCA_002531995.1	I	3	8,941,773
CCBAU 15544	GCA_000261705.1	I	4	8,484,383

CCBAU 15615	GCA_000261725.1	I	4	8,852,539
CCBAU 15635	GCA_000261745.1	I	4	8,288,715
USDA 4	GCA_000472725.1	I	4	8,737,671
OO99[T]	GCA_002278135.1	I	4	8,677,102
L2	GCA_002532025.1	I	4	8,797,357
CCBAU 05623	GCA_000261785.1	I	5	8,245,751
CCBAU 25021	GCA_000261805.1	I	5	7,990,949
CCBAU 35157	GCA_000261825.1	I	5	7,854,129
BR3267	GCA_001439885.1	I	5	7,904,309
SUTN9-2	GCA_003122645.1	I	5	8,092,255
CCBAU 10071[T]	GCA_900094575.1	I	5	8,201,522
WSM2254	GCA_000472645.1	I	6	9,086,516
JGI 0001019-J21	GCA_000485255.1	I	6	1,191,681[c]
cf659	GCA_900113725.1	I	6	8,465,016
CCBAU 05525	GCA_000261645.1	I	7	8,164,124
CCBAU 83689	GCA_000261665.1	I	7	8,009,819
USDA 135	GCA_000472945.1	I	7	7,703,324
USDA 3384	GCA_000472685.1	I	8	9,800,269
CB756	GCA_000473125.1	I	8	9,826,880
LMG 26795[T]	GCA_900116675.1	I	8	9,793,799
is5	GCA_000421305.1	I	9	7,588,794
in8p8	GCA_000426845.1	I	9	7,589,915
Leaf396	GCA_001424605.1	I	9	7,709,278
WSM1417	GCA_000515415.1	I	10	8,048,963
URHA0013	GCA_000518345.1	I	10	7,193,102
WSM2793	GCA_000379585.1	I	11	8,871,253
Rc3b	GCA_900114915.1	I	11	8,375,670
CCGE-LA001	GCA_000296215.2	I	12	7,833,499
DOA1	GCA_001593325.1	I	12	7,543,105
AC87j1	GCA_002936975.1	I	13	8,683,180
ERR11[T]	GCA_900094605.1	I	13	9,163,226
WSM471	GCA_000244915.1	I	14	7,784,016
BF49_genome1	GCA_900011245.1	I	14	7,547,693
Cp5.3	GCA_000426265.1	I	15	8,918,781
BR 10247[T]	GCA_001641695.1	I	15	8,679,329
LTSP849	GCA_000938255.1	I	16	8,570,160
LTSP857	GCA_000938285.1	I	16	8,426,077
OK095	GCA_900109945.1	I	17	7,846,124
Y36	GCA_002531575.1	I	18	7,327,521
S23321	GCA_000284275.1	I	19	7,231,841
TSA1[T]	GCA_002776695.1	I	20	8,199,267
39S1MB	GCA_002266435.1	I	21	7,068,339
UBMA197	GCA_002108935.1	I	22	10,442,239

Ec3.3	GCA_000472925.1	I	23	10,034,502
JGI 0001019-M21	GCA_000485335.1	I	24	236,340[c]
Ghvi	GCA_900115265.1	I	25	7,925,674
INPA54B[T]	GCA_002795245.1	I	26	8,252,029
85S1MB	GCA_002266465.1	I	27	7,037,054
DOA9	GCA_000617845.2	I	28	7,850,549
CCH5-F6	GCA_001556045.1	I	29	8,163,151
BR 10245[T]	GCA_001641635.1	I	30	10,111,113
22	GCA_000482425.1	I	31	7,504,559
CCNWSX0360	GCA_001595995.1	I	32	8,590,559
WSM3983	GCA_000473045.1	I	33	8,789,602
CCBAU 43298	GCA_000261685.1	I	34	7,916,986
NAS80.1	GCA_001908205.1	I	35	9,235,468
Rc2d	GCA_900099855.1	I	36	9,578,216
YR681	GCA_000282615.1	I	37	7,831,714
WSM1253	GCA_000244935.1	I	38	8,719,808
BR10280[T]	GCA_002068095.1	I	39	8,698,798
JGI 0001002-A22	GCA_000464155.2	I	40	1,961,262[c]
BR 446[T]	GCA_001641335.1	I	41	8,801,717
Gha	GCA_900113735.1	I	42	8,253,313
USDA 124	GCA_000374205.1	I	43	9,002,274
MOS002	GCA_003020115.1	I	44	7,575,111
AS23.2	GCA_001908185.1	I	45	8,705,449
BR3351[T]	GCA_001440035.1	I	46	9,145,311
WSM1743	GCA_000473065.1	I	47	8,341,956
AT1	GCA_001590795.1	I	48	7,534,837
MOS003	GCA_003020125.1	I	49	7,261,081
587	GCA_000257685.2	II	50	8,673,157
CCBAU 05737	GCA_000261505.1	II	50	9,773,178
CCBAU 43297	GCA_000261525.1	II	50	9,347,700
USDA 76[T]	GCA_000379145.1	II	50	9,484,767
USDA 94	GCA_000519225.1	II	50	9,558,895
BLY6-1	GCA_001718185.1	II	50	9,202,572
BLY3-8	GCA_001718205.1	II	50	9,198,916
TnphoA 33	GCA_001868735.1	II	50	9,529,806
USDA 3254	GCA_000472765.1	II	51	8,979,722
USDA 3259	GCA_000473005.1	II	51	8,722,461
PAC 48[T]	GCA_001189245.1	II	51	8,711,973
BR3262	GCA_001440015.1	II	51	8,965,178
UFLA 03-321	GCA_001969825.1	II	51	8,595,048
R5	GCA_900101405.1	II	51	9,444,915
OHSU_III	GCA_000497675.1	II	52	7,935,543
UASWS1015	GCA_000878305.1	II	52	7,820,754

UASWS1016	GCA_001705105.1	II	52	7,960,052
UBA2491	GCA_002341725.1	II	52	7,248,648
SK17	GCA_002831585.1	II	52	8,288,568
DFCI-1	GCA_000465325.1	II	53	7,646,147
17-4 str. JCM 18382	GCA_001312945.1	II	53	7,106,372
PARBB1	GCA_002256335.1	II	53	7,722,922
MOS004	GCA_003020075.1	II	53	7,574,276
SEMIA 690^T	GCA_001238275.1	II	54	8,811,922
UFLA03-84	GCA_002289535.1	II	54	8,628,932
LTSPM299	GCA_000938235.1	II	55	9,094,432
LTSP885	GCA_000938305.1	II	55	7,851,216
MT12	GCA_900105845.1	II	56	8,967,172
SEMIA 6208^T	GCA_001189235.2	II	57	8,267,832
SEMIA 6148^T	GCA_001189845.1	II	58	9,767,314
BR 10303^T	GCA_001542415.1	II	59	8,722,143
C9	GCA_002532045.1	II	60	8,428,389
NAS96.2	GCA_001908235.1	II	61	8,927,485
SEMIA 6399^T	GCA_001982635.1	II	62	8,842,857
th.b2	GCA_000426785.1	II	63	10,118,060
ORS 285	GCA_000239755.2	III	64	7,602,254
ORS 285	GCA_900176205.1	III	64	7,797,098
ORS 375	GCA_000239775.2	III	65	7,859,468
STM 3809	GCA_000239795.2	III	66	7,311,748
BTAi1	GCA_000015165.1	III	67	8,493,513
STM 3843	GCA_000239815.2	III	68	8,434,788
S58^T	GCA_000344805.1	III	69	8,264,165
ORS 278	GCA_000026145.1	III	70	7,456,587
GAS524	GCA_900099825.1	IV	71	8,339,215
GAS522	GCA_900104755.1	IV	71	8,269,569
MT34	GCA_900142345.1	IV	71	8,150,968
RST89^T	GCA_003062285.1	IV	72	8,824,342
RST91	GCA_003062295.1	IV	72	8,907,982
LmjM6	GCA_001440385.1	IV	73	9,146,476
LmjM3^T	GCA_001440405.1	IV	73	8,845,258
URHA0002	GCA_000426105.1	IV	74	6,972,827
LMTR 21^T	GCA_001693515.1	IV	75	8,290,608
PAC68^T	GCA_001440395.1	IV	76	8,738,846
URHD0069	GCA_000701345.1	IV	77	6,793,945
CCBAU 23086^T	GCA_001440475.1	IV	78	8,817,291
GAS138	GCA_900129425.1	IV	79	9,092,136
GAS165	GCA_002797515.1	IV	80	6,132,387
GAS478	GCA_900129675.1	IV	81	11,738,562
GAS242	GCA_900129505.1	IV	82	9,184,751

GAS499	GCA_900141755.1	IV	83	7,910,099
LMTR 3	GCA_001693485.1	IV	84	7,834,745
WSM1741	GCA_000472965.1	IV	85	7,952,346
GAS369	GCA_900105125.1	IV	86	7,842,044
LMTR 13[T]	GCA_001693385.1	IV	87	8,322,773
Ro19[T]	GCA_001440415.1	IV	88	8,466,225
ARR65	GCA_000472385.1	V	89	8,613,869
Tv2a-2	GCA_000472425.1	V	90	8,496,279
Ai1a-2	GCA_000426245.1	VI	91	9,029,266
WSM2783	GCA_000472865.1	VI	92	9,902,361
GAS401	GCA_900142985.1	VII	93	7,525,217
CCH1-B1	GCA_001556485.1	-[d]	94	4,299,406
CCH5-A9	GCA_001556725.1	-[d]	94	4,713,629
CCH4-A6	GCA_001556245.1	-[d]	95	5,299,950
CCH10-C7	GCA_001556255.1	-[d]	95	5,592,688
35-63-5	GCA_002280915.1	-[d]	96	515,174
HPC(L)	GCA_000304595.1	-[d]	97	5,272,243
NFR13	GCA_900114605.1	-[d]	98	6,226,103

[a]Based on a phylogenic analysis performed with AMPHORA2.

[b]Based on a 95% cutoff ANI value.

[c]Partial genome.

[d]Does not belong to the *Bradyrhizobium* genus.
