# Supplementary material for: A Genomotaxonomy View of the Bradyrhizobium Genus
Source: Front Microbiol. 2019 Jun 13;10:1334. doi: 10.3389/fmicb.2019.01334 (PMC6585233; doi:10.3389/fmicb.2019.01334)
Supplement: Supplementary file 4 [file Table_2.pdf]

Supplementary Table 2. Presence of genes for the plasmid partition protein RepB in the genomes of *Bradyrhizobium*

**Strain**	**Number of *repB* genes**
Bradyrhizobium liaoningense CCBAU 05525	6
Bradyrhizobium diazoefficiens NK6	5
Bradyrhizobium japonicum USDA 123	4
Bradyrhizobium japonicum USDA 135	3
Bradyrhizobium sp. Leaf396	2
Bradyrhizobium algeriense RST89	2
Bradyrhizobium algeriense RST91	2
Bradyrhizobium sp. Ai1a 2	2
Bradyrhizobium japonicum UBMA197	2
Bradyrhizobium sp. CCH5 F6	2
Bradyrhizobium japonicum 22	2
Bradyrhizobium yuanmingense CCBAU 10071	2
Bradyrhizobium sp. UASWS1016	2
Bradyrhizobium japonicum is5	1
Bradyrhizobium sp. WSM4349	1
Bradyrhizobium sp. AC87j1	1
Bradyrhizobium viridifuturi SEMIA 690	1
Bradyrhizobium sp. Cp5 3	1
Bradyrhizobium elkanii USDA 76	1
Bradyrhizobium elkanii USDA 94	1
Bradyrhizobium elkanii USDA 3254	1
Bradyrhizobium sp. R5	1
Bradyrhizobium sp. Ghvi	1
Bradyrhizobium japonicum USDA 4	1
Bradyrhizobium sp. DOA9	1
Bradyrhizobium sp. BTAi1	1
Bradyrhizobium liaoningense CCNWSX0360	1
Bradyrhizobium sp. th b2	1
Bradyrhizobium sp. WSM1253	1
Bradyrhizobium sp. SK17	1
Bradyrhizobium elkanii WSM2783	1

Bradyrhizobium sp. DFCI 1	1
Bradyrhizobium sp. MOS004	1
Bradyrhizobium sp. Gha	1
Bradyrhizobium manausense BR3351	1
