# Supplementary material for: A Genomotaxonomy View of the Bradyrhizobium Genus
Source: Front Microbiol. 2019 Jun 13;10:1334. doi: 10.3389/fmicb.2019.01334 (PMC6585233; doi:10.3389/fmicb.2019.01334)
Supplement: Supplementary file 2 [file Image_2.pdf]

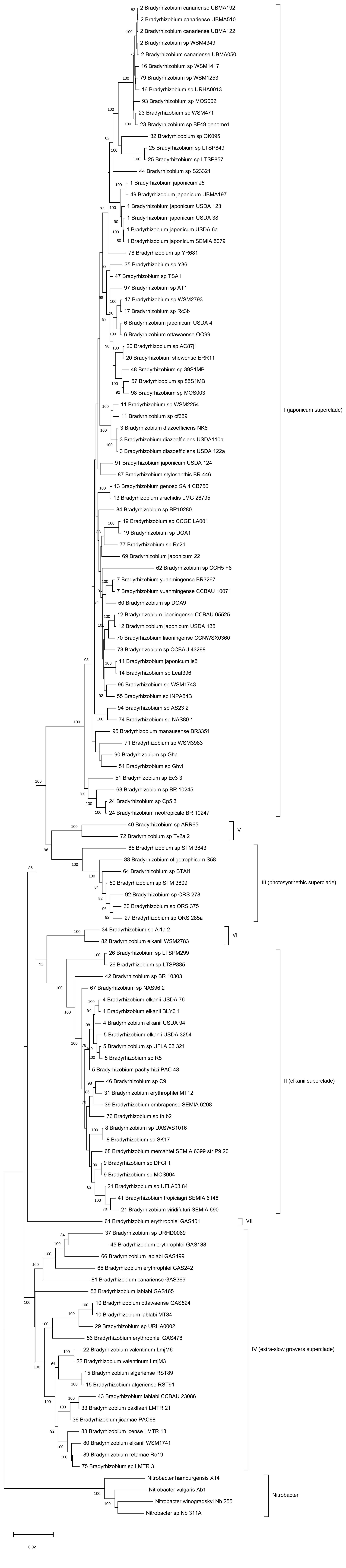

Supplementary Figure 2

Phylogenomic tree of Bradyrhizobium. Superclades are indicated with Roman numerals. The tree was constructed with maximum likelihood using a concatenated alignment of 31 conserved proteins identified with AMPHORA2 (7672 aa alignment length). Bootstrap node support values lower than 70% are not shown.
